# PAC1 deficiency reduces chondrogenesis in atherosclerotic lesions of hypercholesterolemic ApoE-deficient mice

**DOI:** 10.1186/s12872-023-03600-5

**Published:** 2023-11-18

**Authors:** C. Blümm, G. A. Bonaterra, H. Schwarzbach, L. E. Eiden, E. Weihe, R. Kinscherf

**Affiliations:** 1 Institute for Anatomy and Cell Biology, Department of Medical Cell Biology, University of Marburg, 35032 Marburg, Germany; 2 Section on Molecular Neuroscience, Laboratory of Cellular and Molecular Regulation, National Institute of Mental Health Intramural Research Program, Bethesda, MD 20814 USA

**Keywords:** Atherosclerosis, Cardiovascular disease, Chondrogenic transcription factor, Collagen, Macrophages, Vascular calcification, Vascular smooth muscle cells

## Abstract

**Background:**

Induction of chondrogenesis is associated with progressive atherosclerosis. Deficiency of the ADCYAP1 gene encoding pituitary adenylate cyclase-activating peptide (PACAP) aggravates atherosclerosis in ApoE deficient (ApoE^−/−^) mice. PACAP signaling regulates chondrogenesis and osteogenesis during cartilage and bone development. Therefore, this study aimed to decipher whether PACAP signaling is related to atherogenesis-related chondrogenesis in the ApoE^−/−^ mouse model of atherosclerosis and under the influence of a high-fat diet.

**Methods:**

For this purpose, PACAP^−/−^/ApoE^−/−^, PAC1^−/−^/ApoE^−/−^, and ApoE^−/−^ mice, as well as wildtype (WT) mice, were studied under standard chow (SC) or cholesterol-enriched diet (CED) for 20 weeks. The amount of cartilage matrix in atherosclerotic lesions of the brachiocephalic trunk (BT) with maximal lumen stenosis was monitored by alcian blue and collagen II staining on deparaffinized cross sections. The chondrogenic RUNX family transcription factor 2 (RUNX2), macrophages [(MΦ), Iba1^+^], and smooth muscle cells (SMC, sm-α-actin) were immunohistochemically analyzed and quantified.

**Results:**

ApoE^−/−^ mice fed either SC or CED revealed an increase of alcian blue-positive areas within the media compared to WT mice. PAC1^−/−^/ApoE^−/−^ mice under CED showed a reduction in the alcian blue-positive plaque area in the BT compared to ApoE^−/−^ mice. In contrast, PACAP deficiency in ApoE^−/−^ mice did not affect the chondrogenic signature under either diet.

**Conclusions:**

Our data show that PAC1 deficiency reduces chondrogenesis in atherosclerotic plaques exclusively under conditions of CED-induced hypercholesterolemia. We conclude that CED-related chondrogenesis occurs in atherosclerotic plaques via transdifferentiation of SMCs and MΦ, partly depending on PACAP signaling through PAC1. Thus, PAC1 antagonists or PACAP agonists may offer therapeutic potential against pathological chondrogenesis in atherosclerotic lesions generated under hypercholesterolemic conditions, especially in familial hypercholesterolemia. This discovery opens therapeutic perspectives to be used in the treatment against the progression of atherosclerosis.

## Background

Atherosclerosis is a chronic inflammatory disease of the intima of arterial vessels causing multifocal plaque development along the arterial tree and is characterized by endothelial dysfunction, lipid deposition, intimal inflammation, necrosis, fibrosis, and calcification [1, 2]. It leads to loss of elasticity coincident with progressive vascular occlusion with reduced blood flow, which might cause, after decades of subclinical course, acute cardiovascular events such as myocardial infarction or stroke [1, 3]. Cardiovascular diseases are the leading cause of death worldwide [4, 5].

Hypercholesterolemic apolipoprotein E deficient (ApoE^−/−^) mice are routinely used to investigate progressive atherosclerosis because they reveal plaques in the brachiocephalic trunk (BT) with features that represent those in vulnerable human plaques [6]. Hypercholesterolemia, low-density lipoprotein (LDL) cholesterol, and especially subendothelial accumulation of oxidatively modified low-density lipoproteins (oxLDL) play a crucial role in the initiation and progression of atherosclerotic lesions [1, 7]. Both atherogenesis and atheroprogression involve the activation of the innate and adaptive immune system and the complex interaction of proinflammatory cytokines and chemokines that increase the size and number of atherosclerotic lesions, which affects the arterial hemodynamic capacity [8]. As the disease progresses, the plaques’ cell types and morphology change. Studies by electron beam tomography demonstrated that 90% of all atherosclerotic lesions of older people show calcified areas [9]. In this context, mineralization and calcification of atherosclerotic lesions are highly regulated and non-spontaneous, multi-step processes [10, 11]. In advanced calcified atherosclerotic lesions, cartilage and bone matrix proteins and chondrocyte- and osteoblast-like cells have been identified in human and animal models [11–15]. Vascular calcification evolves from macrophage (MΦ)-mediated inflammation, as well as from the osteochondrogenic transition of smooth muscle cells (SMCs) in atherosclerotic lesions [16]. Moreover, SMCs in atherosclerotic lesions were shown to transdifferentiate from a differentiated contractile to an undifferentiated proliferative, chondrocyte-like phenotype, characterized by expression of cartilage and bone matrix proteins as well as chondroid transcriptions factors [17–23]. This phenotypic modulation of SMCs to “chondromyocytes” is suggested to be regulated by, e.g., the aryl hydrocarbon receptor RUNX family transcription factor 2 (RUNX2), etc. [24, 25]. Injury as mechanical or oxidative stress and expression of RUNX2 are involved in the osteogenic differentiation of SMCs [26, 27]. When the expression of RUNX2 is increased in the tunica media of the arterial wall, SMCs differentiation to osteoblast and calcification are stimulated [25, 28].

Pituitary adenylate cyclase-activating polypeptide (PACAP) is a widely distributed neurotransmitter of the central and peripheral nervous system and mediates pleiotropic anti-inflammatory, immunomodulating, cytoprotective, and anti-proliferative effects [29, 30]. PACAP acts in various physiological and pathophysiological processes, including neuroprotection, neurodegeneration, pain, energy metabolism, respiratory and cardiac functions and dysfunctions [31]. PACAP is a crucial regulator of neuroendocrine stress circuits via the hypothalamic-pituitary-adrenal and the splanchnic-adrenomedullary axes [32]. PACAP exerts its effects through three heterotrimeric G protein-coupled receptors [33–36]. The PAC1 receptor binds PACAP nearly selectively, whereas the vasoactive intestinal polypeptide receptor 1 (VPAC1) and vasoactive intestinal polypeptide receptor 2 (VPAC2) receptors show similar binding affinities for both PACAP and vasoactive intestinal peptide (VIP) [37, 38]. Recent evidence shows PACAP deficiency aggravates atherosclerosis in ApoE^−/−^ mice [39]. Therefore, PACAP has been suggested to act as an endogenous athero-protective neuropeptide [39], whereas PAC1 deficiency attenuates the progression of atherosclerosis in ApoE^−/−^ mice [40]. Moreover, PACAP and PAC1 are expressed in chondroid cell cultures and PACAP enhances the cartilage matrix production and is involved in regulating extravascular chondrogenesis and osteogenesis (41, 42). As chondrogenesis is part of atherosclerotic vascular remodelling the question arises whether vascular chondrogenesis during atherogenesis is influenced by endogenous PACAP signalling. Therefore, we aimed to explore the influence of PACAP deficiency or PAC1 deficiency on vascular chondrogenesis in atherosclerotic plaques in ApoE^−/−^ mice fed with standard chow (SC) or cholesterol-enriched diet (CED).

## Methods

### Animals

ApoE^−/−^ mice (Charles River, Sulzfeld, Germany) were crossbred with PACAP knockout (ADCYAP1^−/−^ / PACAP^−/−^) [32] or PAC1 knockout (PAC1^−/−^) [43] mice to generate PACAP^−/−^/ApoE^−/−^ and PAC1^−/−^/ApoE^−/−^ mice. In addition to male, homozygous ApoE^−/−^, PACAP^−/−^/ApoE^−/−^, and PAC1^−/−^/ApoE^−/−^ mice, male wildtype (WT) mice were included in the study. The groups of mice were randomly assigned. At the age of 10 weeks, all mice were fed either SC (LASQCdiet® Rod16 Rad; LASvendi, Soest, Germany) or CED “western type diet” [21% fat, 0.15% cholesterol and 19.5% casein], Altromin GmbH, Lage, Germany) for 20 weeks. The mice were housed in 4 to 5 in each cage under the same conditions, with dark–light cycles of 12 h and temperature of 24 ± 2 °C with ad libitum access to food and water. The animals were kept in cages with an area of 100 cm^2^ per animal according to the GVSOLAS (Committee for Animal Welfare Laboratory animal husbandry, August 2014. https://www.gv-solas.de/wp-content/uploads/2021/08/hal_201408Tiergerechte-Haltung-Maus.pdf) with appropriate environmental enrichment. Approval or permission from the farm owner to use the animals was unnecessary. The Regional Commission Gieβen approved the study (V54-19 c 2015 h 01 MR 20/26 Nr. 21/2014) following the regulations for animal experimentation of the Philipps-University of Marburg. Our manuscript adheres to the ARRIVE guidelines (http://www.nc3rs.org.uk/page.asp?id=1357) for the reporting of animal experiments.

### Genotyping

Genomic deoxyribonucleic acid (DNA) was isolated from an ear biopsy using a commercial kit (DNA Extraction Solution; PeqLab, VWR Company, Erlangen, Germany) following the manufacturer’s instructions (DirectPCR® lysis reagent ear Peqlab, VWR International; Darmstadt, Germany). Afterward, homozygous transgenic mice were identified by polymerase chain reaction (PCR) using intron-spanning oligonucleotides [32, 39]. PCR analysis showed the characteristic single bands for the WT alleles, PACAP^**−/−**^ (310 bp), PAC1^**−/−**^ (265 bp), and ApoE^**−/−**^ (245 bp) (data not shown).

### Dissection and tissue harvesting

The 30 weeks old mice were weighed and anesthetized with a combination of ketamine (150 mg/kg) and xylazine (20 mg/kg). Subsequently, the thoracic cavity was opened through a thoracotomy, the left ventricular apex was incised and a cannula (8G, B. Braun Melsungen AG, Melsungen, Germany) was introduced and clamped. After the incision of the right atrium, blood samples were taken. The vascular system was perfused with a solution of 39 °C warmed phosphate-buffered saline (PBS) and 5 Ul/ml heparin (Liquemin® 25,000 Ul/5ml, Roche, Grenzach, Germany), at the rate of 100 ml/h and a total volume of 30 ml using an automated syringe-pump (Secura, B. Braun, Melsungen, Germany). After this, the vascular system was perfused with 200–300 µl filtered isotonic sodium chloride solution containing methylene blue (0.25%; Riedel-de Haën, Seelze-Hannover, Germany). Afterward, the brachiocephalic trunk (BT) was excised utilizing a binocular loupe, fixed in paraformaldehyde (PFA) 4% in PBS, dehydrated through graded ethanols, and embedded in paraffin for immunohistomorphometrical analyses.

### Determination of plasma lipid levels

At the point of opening the right atrium, blood samples were taken. Blood samples were heparinized (0.25 IU/ml), centrifuged (10 min, 650 x g) and the supernatant plasma was separated and stored at -80ºC. Plasma total cholesterol and triglyceride- levels were determined spectrophotometrically using a microplate reader (Sunrise, Tecan, Männedorf, Switzerland) and commercially available kits (cholesterol/cholesteryl ester quantitation kit ab65359, or triglyceride quantification assay kit ab65336, Abcam, Cambridge, UK).

### Morphometry and immunohistology

Since the extension of each plaque is not unlimited, the BT region with maximal lumen stenosis was detected by serial cross-Sect. (6 μm) of paraffin-embedded BT. Subsequently a standard hematoxylin-eosin (HE) staining and quantification of lumen stenosis was performed every 72 μm (approximately 700–1500 μm total BT length). Cross-sections with maximal lumens stenosis or directly proximally or distally localized beside were used for immunohistochemical and histomorphometrical analyses, i.e. one cross-section for every immunohistomorphometrical analysis per mouse. For this purpose, the lumen and plaque areas were traced along the internal elastic lamina, and lumen stenosis was calculated [(plaque area [µm^2^]) / (lumen area [µm^2^]) x 100%=lumen stenosis (%)]. For the subsequent investigations, cross-sections in maximal lumen stenosis were chosen. Alcian blue and immunohistochemical (IHC) stains were performed to analyze chondrogenesis within the arteriosclerotic lesions, as described earlier [44, 45], using antibodies as listed in Table 1. Previously sodium citrate retrieval was performed at 92° to 95 °C (10 min). The quantification of the immunoreactive (IR) plaque areas was assessed similarly to the quantification of the lumen stenosis by tracing the IR and the total plaque areas [(IR plaque area [µm^2^]) / (total plaque area [µm^2^]) x 100%= IR plaque area (%)]. The evaluation (in %) of the RUNX2 immunoreactive (IR) nuclei was performed by analyzing the proportion of IR nuclei within the atherosclerotic plaque to all nuclei of the plaque.

**Table 1:** Antibodies used in this study

Name	Company	Dilution
Donkey anti-rabbit IgG biotin-conjugated	Jackson ImmunoResearch, West Grove, USA	1:200
Rabbit anti-mouse smooth muscle α actin	Abcam, Cambridge, UK	1:500
Rabbit anti-mouse ionized calcium-binding Ionized Calcium-Binding Adapter Molecule 1 (Iba1)	Wako Pure Chemical Industries, Osaka, Japan	1:1000
Rabbit anti-mouse collagen II	Abcam, Cambridge, UK	1:400
Rabbit anti-mouse runt-related transcription factor 2 (RUNX2)	Abcam, Cambridge, UK	1:50

### Statistical analyses

All statistical analyses were performed using SigmaPlot 12 (Systat Software Inc., San José, USA). The data was analyzed using the Shapiro-Wilk normality and Brown-Forsythe equal variance tests. Statistical significance of normally distributed data was determined by the unpaired two-tailed t-test, in case data failed the normality test, by the Mann-Whitney rank-sum test. In case of failed equal variance test, Welch’s t-test was applied. A two-way analysis of variance (ANOVA) was used to examine the interaction between independent variables. Results are shown as mean values + standard error of the mean (SEM). Differences were considered statistically significant for p < 0.05.

## Results

### PAC1 deficiency reduces the body weight of ApoE-deficient mice under SC

The body weight of PAC1^-/-^/ApoE^-/-^ mice under SC (30 wks) was 4.5 g (p = 0.015) lower than that of ApoE^-/-^ mice, whereas the body weight of PACAP^-/-^/ApoE^-/-^ and ApoE^-/-^ mice under SC was almost identical (Table 2). After CED, no significant differences in body weight were found between PACAP^-/-^/ApoE^-/-^ and PAC1^-/-^/ApoE^-/-^ or ApoE^-/-^ mice (Table 2). After SC, ApoE^-/-^ mice revealed a 4.2 g (p = 0.032) lower body weight than WT mice, and after CED, a 4.0 g (p = 0.010) higher body weight than WT mice (Table 2). After CED, PAC1^-/-^/ApoE^-/-^ mice were 9.1 g (p = 0.007) heavier than PAC1^-/-^/ApoE^-/-^ mice that received SC (Table 2).

The tibia lengths of ApoE^-/-^, PACAP^-/-^/ApoE^-/-^ and PAC1^-/-^/ApoE^-/-^ mice were similar after SC and CED (Table 2). These data are in line with our previously published observations [39, 40].

**Table 2:** Effect of PACAP and PAC1 deficiency on body weight, tibia length, plasma cholesterol, and triglyceride levels

	Wildtype (n = 5–6)	ApoE^−/−^ (n = 4–5)	PACAP^−/−^/ApoE^−/−^ (n = 5–8)	PAC1^−/−/^ApoE^−/−^ (n = 4–7)
**Body weight (g)**				
30 wks SC	35.8 ± 2.1	31.6 ± 0.3*	31.2 ± 0.7	27.1 ± 1.3^§^
10 wks SC + 20 wks CED	29.9 ± 0.8	33.9 ± 1.1**	39.7 ± 3.0	36.2 ± 1.6
**P < vs 30 wks SC**	^##^	N.S.	N.S	^##^
**Tibia length (mm)**				
30 wks SC	18.3 ± 0.4	19.0 ± 0.7	17.9 ± 0.3	18.0 ± 0.9
10 wks SC + 20 wks CED	18.9 ± 0.3	19.2 ± 0.9	19.5 ± 0.2	20.1 ± 0.3
**P < vs 30 wks SC**	N.S	N.S.	^###^	^#^
**Cholesterol (mg/dl)**				
30 wks SC	109.1 ± 5.2	667.0 ± 53.2*	517.7 ± 42,0	413.2 ± 103.9
10 wks SC + 20 wks CED		1040.7 ± 257.1**	999.3 ± 257,1	884.5 ± 206.1
**P < vs 30 wks SC**	67.8 ± 13.0 ^#^	N.S.	^##^	N.S.
**Triglyceride (mg/dl)**				
30 wks SC	47.7 ± 11.2	87.2 ± 6.3*	133.0 ± 28.4	109.5 ± 23.0
10 wks SC + 20 wks CED		97.7 ± 12.4*	134.0 ± 14.8	258,8 ± 79.6
**P < vs 30 wks SC**	60.0 ± 8.9 N.S.	N.S.	N.S.	N.S.

ApoE indicates apolipoprotein E; CED, cholesterol-enriched diet; PACAP, pituitary adenylate cyclase-activating polypeptide; PAC1, pituitary adenylate cyclase-activating polypeptide type I receptor; SC, standard chow; wks, weeks; WT, wildtype. * p < 0.05; ** p < 0.01; *** p ≤ 0.001 vs. wildtype; ^§^p < 0.05 vs. ApoE^−/−^; ^#^p < 0.05; ^##^ p < 0.01; ^###^ p ≤ 0.001 vs. 30 wks SC; N.S. not significant

### Neither PACAP nor PAC1 deficiency affects plasma cholesterol and triglyceride levels

PACAP^-/-^/ApoE^-/-^, PAC1^-/-^/ApoE^-/-^ and ApoE^-/-^ mice revealed 4-6-fold (SC) or 15.3-fold (CED) increased plasma cholesterol levels compared to WT mice (Table 2). ApoE^-/-^ mice had a 1.8-fold (p = 0.042) (SC) or 1.6-fold (p = 0.034) (CED) increase in plasma triglyceride levels compared to WT mice (Table 2). However, plasma cholesterol and triglyceride levels of PACAP^-/-^/ApoE^-/-^, PAC1^-/-^/ApoE^-/-^ and ApoE^-/-^ mice showed no significant differences after SC or CED (Table 2). These blood lipid concentrations are in essential accordance with our previously published observations [39, 40]. PAC1 deficiency reduces glycosaminoglycans within atherosclerotic plaques in BT of ApoE^-/-^ mice under CED.

**Fig. 1:**
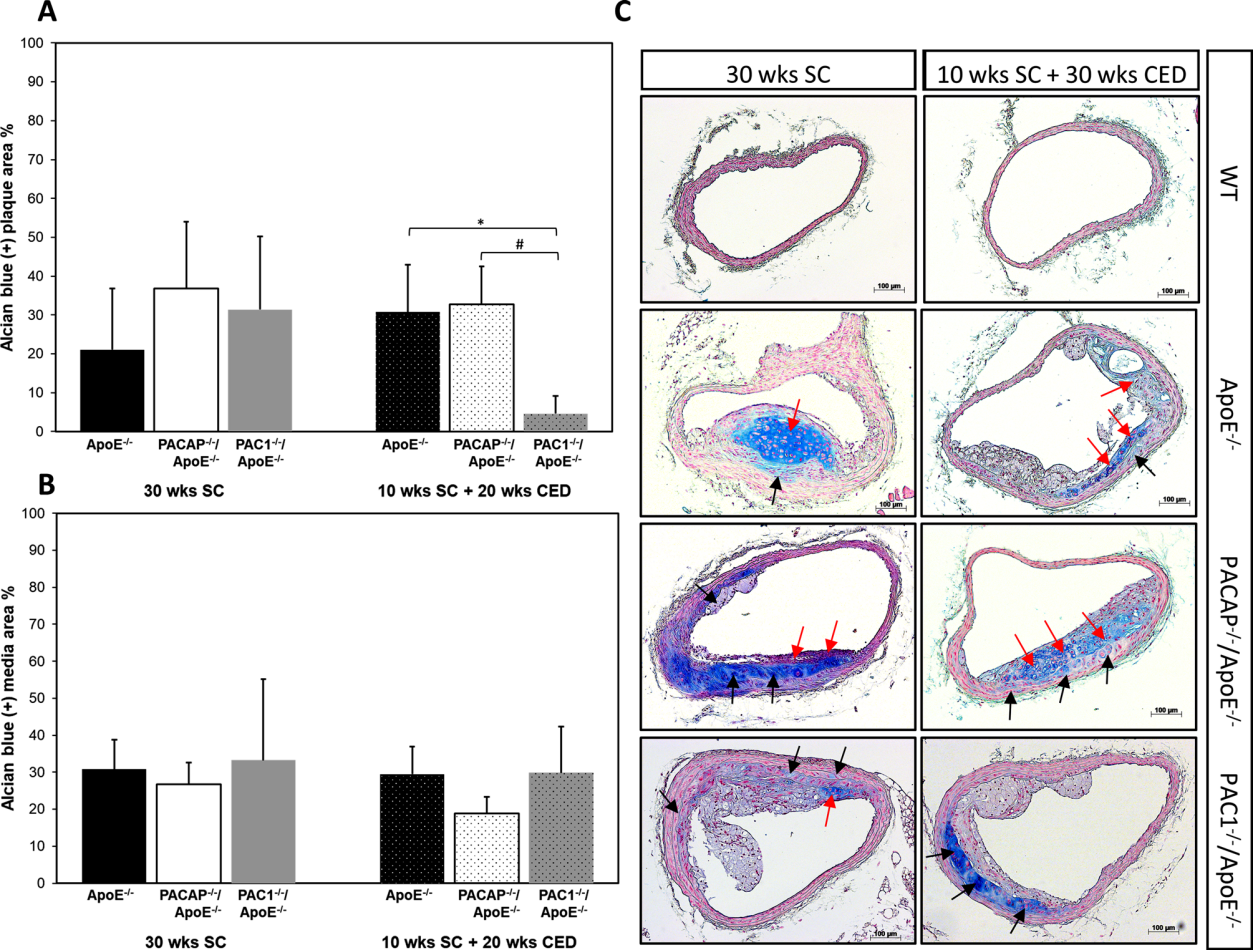
Effect of PACAP- or PAC1 deficiency on the amount of cartilage matrix identified by alcian blue positive glycosaminoglycans in atherosclerotic plaques in the BT. Glycosaminoglycans observed in atherosclerotic plaques (**A**) and neighboring media (**B**) in ApoE^−/−^, PACAP^−/−^/ApoE^−/−^ and PAC1^−/−^/ApoE^−/−^ mice after 30 weeks SC or 10 weeks SC and 20 weeks CED. (**C**) Representative cross-sections after alcian blue staining. Red arrows indicate positive reaction within the intima, and black arrows indicate positive reaction within the media. The data show means + SEM; significance: *p < 0.05 ApoE^−/−^ vs. PAC1^−/−^/ApoE^−/−^ ; ^##^p < 0.05 PAC1^−/−^/ApoE^−/−^ vs. PACAP^−/−^/ApoE^−/−^. Scale bar 100 μm. N = 3–8

Alcian blue allows the detection of extracellular matrix (ECM) glycosaminoglycans and presumptive cartilage due to high concentrations of high molecular weight glycoprotein. After 30 weeks of SC, no alcian blue^+^ plaque area differences in BT were observed among the three ApoE^-/-^ genotypes (Fig. 1A C). In contrast, under CED, we found a significant (p = 0.027) 28.2% reduction of the alcian blue^+^ plaque area in PAC1^-/-^ApoE^-/-^ mice compared to PACAP^-/-^ApoE^-/-^, as well as a significant (p = 0.046) 26.1% lower alcian blue^+^ plaque area than in ApoE^-/-^ mice (Fig. 1A C). PACAP^-/-^/ApoE^-/-^ showed no significant differences in alcian blue^+^ plaque area in BT compared to ApoE^-/-^ mice (Fig. 1A C). Within tunica media areas beneath intimal atherosclerotic lesions, the quantitative analyses revealed no differences among the three ApoE^-/-^ genotypes and feeding (Fig. 1B).

### Neither PACAP nor PAC1 deficiency altered the amount of collagen II within the atherosclerotic lesions in BT of ApoE^-/-^ mice

**Fig. 2:**
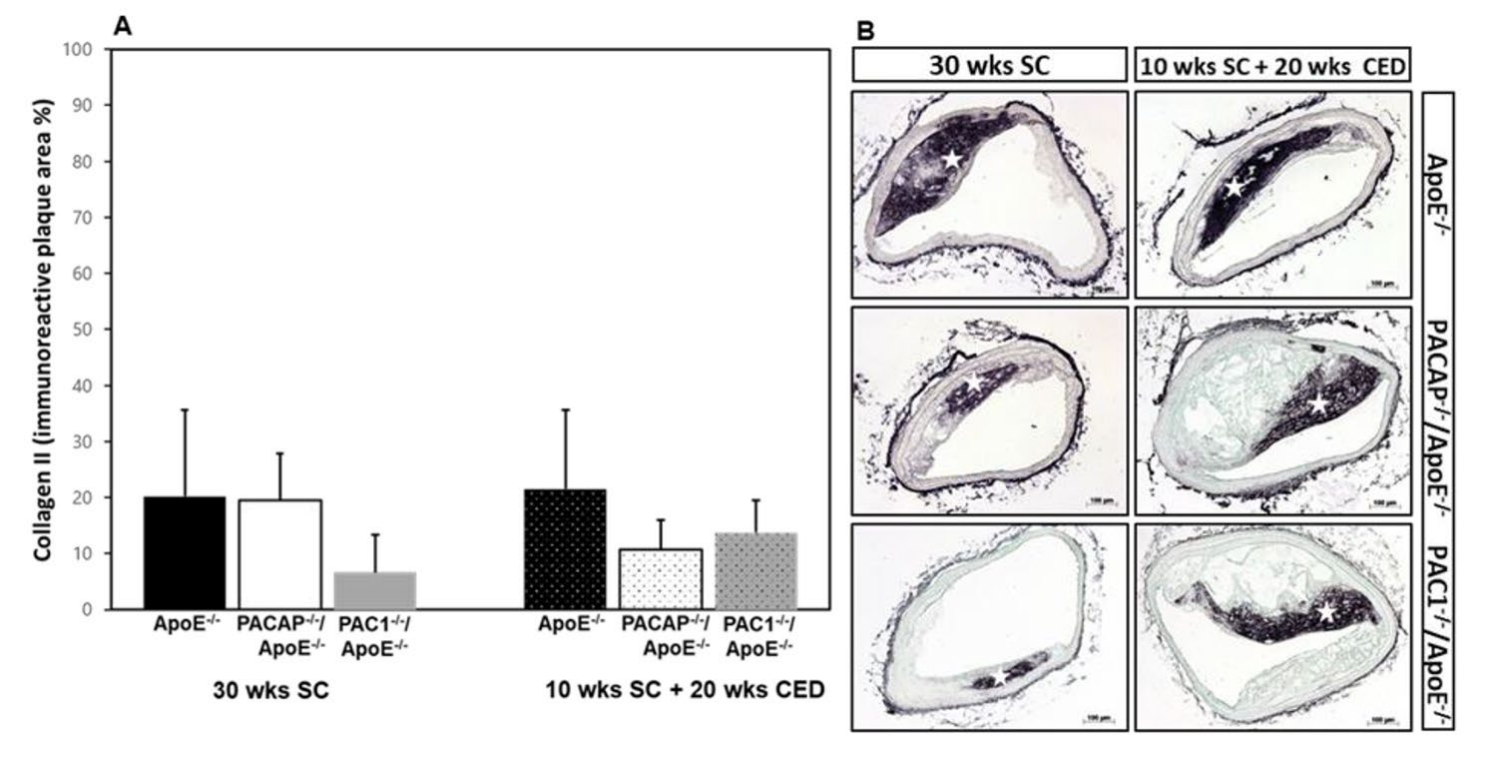
Influence of PACAP- or PAC1 deficiency on cartilage matrix in atherosclerotic plaques in the BT. Histomorphometric analysis of atherosclerotic lesions was performed in ApoE^−/−^, PACAP^−/−^/ApoE^−/−^ and PAC1^−/−^/ApoE^−/−^ mice after 30 weeks SC or 10 weeks SC and afterward 20 weeks CED. Analyses of collagen II^+^ IR plaque areas (**A**) on deparaffinized cross-sections of the BT (**B**) Representative photomicrographs, white stars indicate collagen II positive areas. The data show means + SEM. Scale bar = 100 μm. N = 3–8

**Fig. 3:**
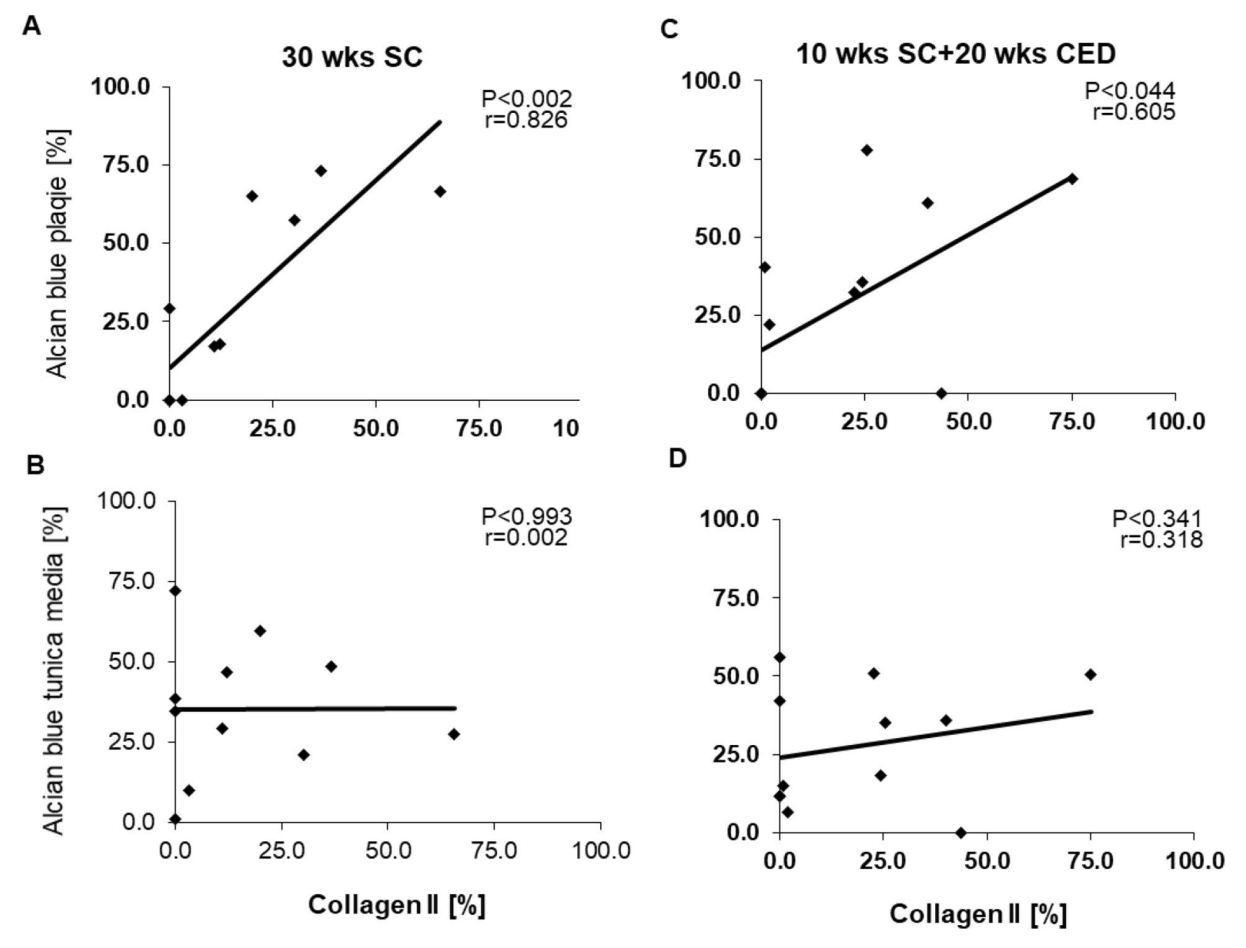
Scatter fit plot curve of Pearson’s correlation analysis between collagen II^+^ IR plaque areas and % alcian blue^+^ plaque area in the atherosclerotic plaques or tunica media, (**A, B**) after 30 weeks SC and (**C, D**) after 10 weeks SC + 20 weeks CED. Abbreviations: r, correlation coefficient; p, P-value; weeks (wks)

Collagen II represents a cartilage-specific protein of the cartilage extracellular matrix. Neither under SC nor under CED were significant differences observed in the collagen II^+^ plaque areas of PACAP^-/-^/ApoE^-/-^, PAC1^-/-^/ApoE^-/-^ and ApoE^-/-^ mice (Fig. 2A). It is noteworthy that the location of collagen II^+^ IR was different in ApoE^-/-^ compared to PACAP^-/-^/ApoE^-/-^, PAC1^-/-^/ApoE^-/-^ mice after 20 weeks CED (Fig. 2B). In ApoE^-/-^ mice collagen II^+^ IR was located inside the plaque, while in the other two genotypes collagen II^+^ was situated in the para-luminal region of the atherosclerotic plaque (Fig. 2B). Significantly positive correlations between collagen II^+^ plaque areas and alcian blue were found in plaques after SC (r = 0.826, p < 0.002) or after CED (r = 0.605, p < 0.044), while no correlations between these two parameters were observed in the tunica media (Fig. 3A and D).

### Influence of PACAP or PAC1 deficiency on the chondroid transcription factor RUNX2 production

**Fig. 4:**
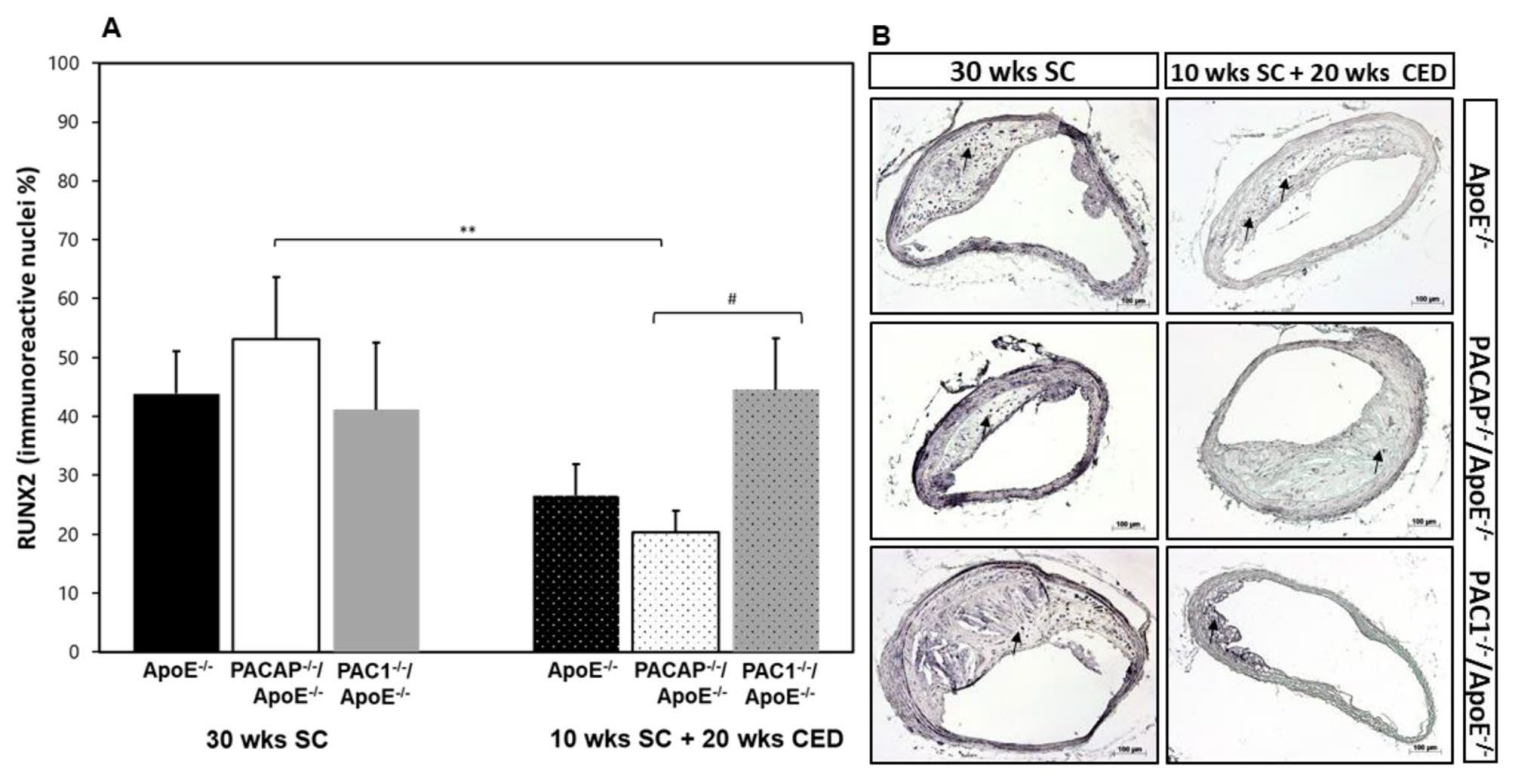
Influence of PACAP- or PAC1 deficiency on chondroid transcription factors in atherosclerotic plaques in the BT. Histomorphometric analysis of atherosclerotic lesions was performed in ApoE^−/−^, PACAP^−/−^/ApoE^−/−^ and PAC1^−/−^/ApoE^−/−^ mice after 30 weeks SC or 10 weeks SC and afterward 20 weeks CED. Analyses of % RUNX2^+^ IR nuclei (**A**), on deparaffinized cross-sections of the BT (**B**) In representative photomicrographs, black arrows indicate a positive reaction. The data show means + SEM; significance: ^**^p < 0.01, PACAP^−/−^/ApoE^−/−^ (30 wks SC) vs. PACAP^−/−^/ApoE^−/−^ (20 wks CED); ^#^p < 0.05, PAC1^−/−^/ApoE^−/−^ vs. PACAP^−/−^/ApoE^−/−^. Scale bar 100 μm. N = 3–8

The transcription factor RUNX2 is a crucial regulator of chondrogenic and osteogenic differentiation. Compared to PACAP^-/-^/ApoE^-/-^ the plaques of PAC1^-/-^/ApoE^-/-^ mice showed a significant (p = 0.033) 24.2% increase in the percentage of RUNX2^+^ nuclei after 20 weeks CED, but not after SC (Fig. 4A and B). However, after CED, PACAP^-/-^/ApoE^-/-^ mice revealed a 32.8% (p = 0.004) lower percentage of RUNX2^+^ nuclei within the plaque compared to PACAP^-/-^/ApoE^-/-^ mice after SC (Fig. 4A).

### Influence of PACAP or PAC1 deficiency on the amount of sm-α-actin^+^ smooth muscle cells in atherosclerotic lesions of ApoE^-/-^ mice

**Fig. 5:**
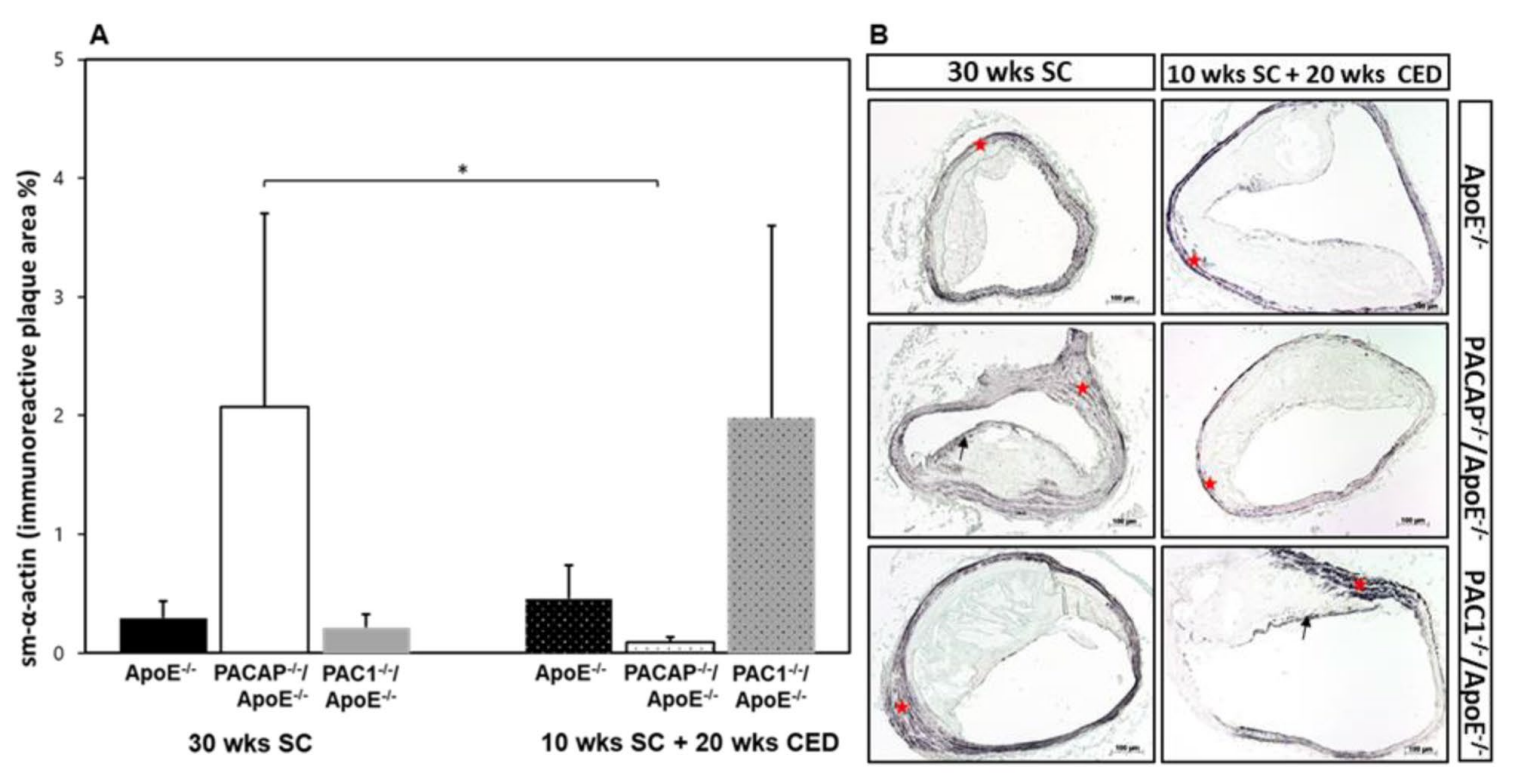
Influence of PACAP- or PAC1 deficiency on sm-α-actin in atherosclerotic plaques in the BT. Histomorphometric analyses of atherosclerotic lesions were performed in ApoE^−/−^, PACAP^−/−^/ApoE^−/−^ and PAC1^−/−^/ApoE^−/−^ mice after 30 weeks SC or 10 weeks SC and afterward 20 weeks CED. Analyses of sm-α-actin^+^ IR plaque area (**A**) on deparaffinized cross-sections of the BT (**B**). In representative photomicrographs, the black arrows indicate a positive reaction within the plaque and red stars in tunica media. The data show means + SEM; significance: ^*^p < 0.05, PACAP^−/−^/ApoE^−/−^ (30 wks SC) vs. PACAP^−/−^/ApoE^−/−^ (20 wks CED). Scale bar 100 μm. N = 3–8

SMCs have an extraordinary capacity to undergo phenotypic changes and adopt features of various mesenchymal cells. In this context, SMCs are suggested as cells of origin of the cartilage-like areas within atherosclerotic lesions. The sm-α-actin^+^ plaque area of PACAP^-/-^/ApoE^-/-^ and PAC1^-/-^/ApoE^-/-^ mice exhibited no significant differences compared to ApoE^-/-^ mice after SC or CED (Fig. 5A and B). However, after CED, PACAP^-/-^/ApoE^-/-^ mice revealed a 2.0% (p = 0.019) lower percentage of sm-α-actin^+^ plaque area within the plaque compared to PACAP^-/-^/ ApoE^-/-^ mice after SC (Fig. 5A and B). It is noteworthy to mention that after 10 weeks SC + 20 weeks CED the sm-α-actin^+^ plaque area correlated significantly positive with the % of RUNX2 positive nuclei (r = 0.719; p < 0.01), and with RUNX2 IR plaque area (r = 0.985, p < 0.001) (Table 3).

**Table 3:** Correlation analyses

		30 wks SC		10 wks SC + 20 wks CED	
		Iba1-Mac	Sm-α actin	Iba1-Mac	Sm-α actin
Alcian Blue^+^ (Area)	*r*	-0.636	0.298	-0.434	-0.287
	*p*	0.035	0.373	0.159	0.365
	*n*	11	11	12	12
RUNX2^+^ (Nuclear)	*r*	-0.705	-0.18	0.312	0.719
	*p*	0.016	0.596	0.323	< 0.01
	*n*	11	11	12	12
RUNX2^+^ (Area)	*r*	0.255	-0.108	-0.075	0.985
	*p*	0.449	0.753	0.817	< 0.001
	*n*	11	11	12	12

Pearson’s correlation coefficient between Iba1^+^ IR plaque area MΦ (Iba1-Mac) or sm-α-actin^+^ IR plaque area (Sm-α actin) and alcian blue^+^ plaque area [Alcian blue^+^ (Area)], % RUNX2^+^ IR nuclei [(RUNX2^+^ (Nuclear)]), or % RUNX2^+^ IR area [(RUNX2^+^ (Area )]). Significant correlations are marked in gray. Abbreviations: SC, standard chow; CED, cholesterol-enriched diet; n, number of animals; r, correlation coefficient; p, P-value; weeks (wks)

### Influence of PACAP or PAC1 deficiency on the amount of Iba1-positive MΦ

**Fig. 6:**
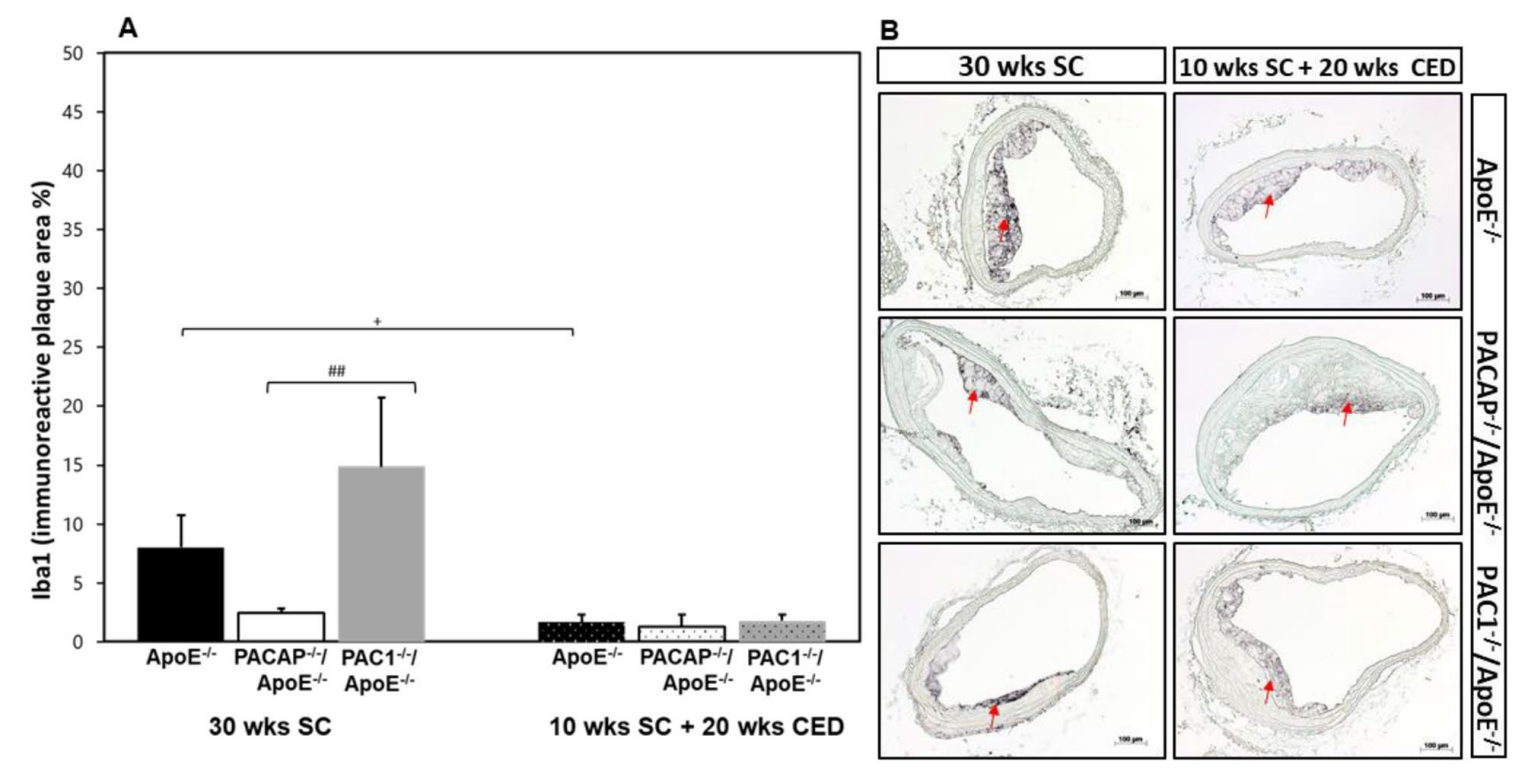
Influence of PACAP- or PAC1 deficiency on MΦ in atherosclerotic plaques in the BT. Histomorphometric analyses of atherosclerotic lesions observed in ApoE^−/−^, PACAP^−/−^/ApoE^−/−^ and PAC1^−/−^/ApoE^−/−^ mice after 30 weeks SC or 10 weeks SC and afterward 20 weeks CED. Analyses of Iba1^+^ IR plaque area MΦ (**A**) on deparaffinized cross-sections of the BT (**B**) In representative photomicrographs, red arrows indicate a positive reaction. The data show means + SEM; significance: ^+^p < 0.05, ApoE^−/−^ (30 wks SC) vs. ApoE^−/−^ (20 wks CED); ^##^p < 0.01, PAC1^−/−^/ApoE^−/−^ vs. PACAP^−/−^/ApoE^−/−^ (30 wks SC). Scale bar 100 μm. N = 3–8

After SC, we found a significant (p = 0.002) 12.4-fold increase of plaque area of Iba1^+^ (ionized calcium-binding adapter molecule 1) MΦ in PAC1^-/-^/ApoE^-/-^ compared to PACAP^-/-^/ApoE^-/-^, but not compared to ApoE^-/-^ mice (Fig. 6A and B). In atherosclerotic plaques of ApoE^-/-^ mice, we measured a significant (p = 0.039) 6.3% decrease of plaque area of Iba1^+^ MΦ after 20 weeks of CED in comparison with SC (Fig. 6A and B).

### Foam cells are positive for RUNX2 and show variable Iba1-immunoreactivity

**Fig. 7:**
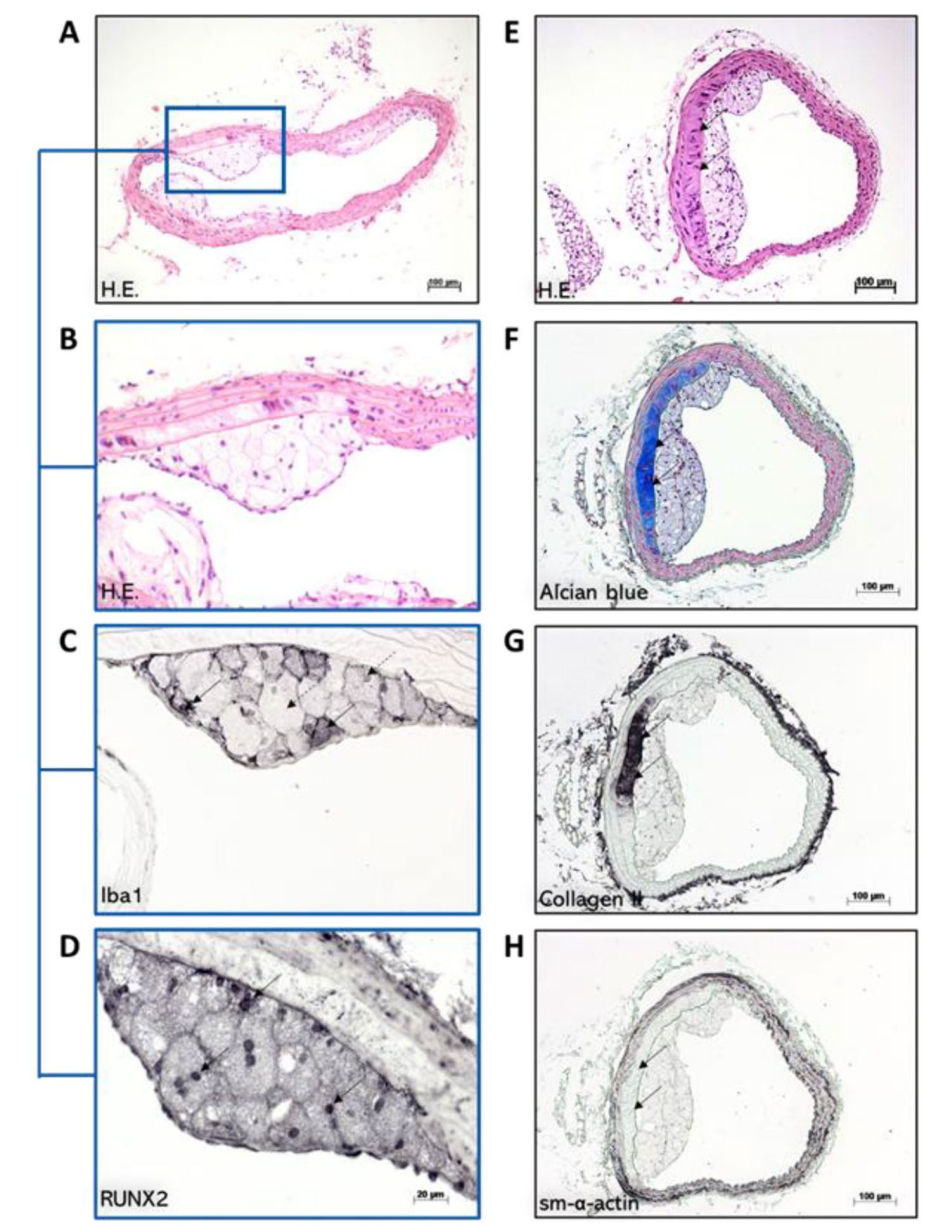
Subpopulations of MΦ and SMC during transdifferentiation processes into osteochondral cells within atherosclerotic plaques in the BT. **A**, **B**, **C**, and **D** show serial cross-sections after HE staining, Iba1- and RUNX2-immunohistochemistry. Foam cells in atherosclerotic lesions show variable immunoreactivity for Iba1 (**C**). Black arrows indicate Iba1-positive foam cells, and dotted arrows indicate Iba1-negative foam cells. Foam cells show positive reactions for RUNX2 (**D**). **E**, **F**, **G**, and **H** show serial cross-sections of the BT after HE- (**E**) and alcian blue staining (**F**), collagen II- (**G**), and SMA immunohistochemistry (**H**). Cartilage-like areas within the media that stain positive for alcian blue and collagen II (**F, G**), are in part not IR for sm-*α-*actin (**H**). Black arrows indicate the affected areas of the neighboring cross-sections. Cartilage-free areas show immunoreactivity for sm-*α-*actin

Qualitative analyses revealed that neighboring foam cells within the atherosclerotic lesion of BT show variable Iba1-IR (Fig. 7A and B, and 7 C). Moreover, foam cell subpopulations in atherosclerotic plaques show RUNX2-immunoreactivity (Fig. 7A and B, and 7D). The percentage of RUNX2 + IR nuclei correlates significantly inversely (p = 0.016, r=-0.705) with Iba1^+^ IR plaque area MΦ after 30 weeks of SC and positively (p < 0.01, r = 0719), after 20 weeks CED (Table 3). After 30 weeks of SC, plaque area of (Iba1)^+^ MΦ correlated significantly inversely with alcian blue plaque area (r=-0.636, p < 0.035). In contrast, such correlations were not observed after 10 weeks SC + 20 weeks CED (Table 3).

### SMCs lose their specific markers in areas of chondroid metaplasia

Qualitative immunohistochemistry and histochemical analyses revealed cartilage-like areas within the media that stain positive for alcian blue and collagen II but not for sm-*α-*actin (Fig. 7E-H). In contrast, neighboring areas free from cartilage-like matrix show immunoreactivity for sm-*α-*actin (Fig. 7H).

## Discussion

The essential new finding of our report is the evidence that PAC1 deficiency inhibits chondrogenesis in the atherosclerotic wall of hypercholesterolemic ApoE knockout mice under CED, but not under SC. Our novel findings are in principle agreement with previous reports of our group demonstrating that PACAP deficiency accelerates and aggravates atherosclerosis in ApoE^−/−^ mice [39], whereas PAC1 deficiency attenuates the progression of atherosclerosis in ApoE deficient mice [40]. However, these results were of major interest for PAC1^−/−^/ApoE^−/−^ mice since the lack of PAC1 inhibited the formation of plaques [40], and exhibited an opposite phenotype to that of PACAP^−/−^/ApoE^−/−^ mice [39]. In our previous publication we have assumed that the anti-atherogenic effect of PACAP was only mediated through the PAC1 receptor [40]. However, PAC1^−/−^/ApoE^−/−^ mice still have endogenous PACAP, which, through its binding to its receptors VPAC1 and/or VPAC2, may also exert an anti-atherogenic effect by skipping the PAC1 way. Thus, it may be possible that PACAP-mediated activation of the PAC1 receptor is not central to the anti-atherogenic effect of PACAP. Yet, it remains to be determined which PACAP receptor/s, VPAC1 and/or VPAC2 alone or together is/are responsible for the observed anti-atherosclerotic effects and possibly also reducing the cartilage formation. However, related to cartilage formation,, our report is the first to show that vascular chondrogenesis like non-vascular chondrogenesis seems to be regulated by PACAP signalling. Regarding non-vascular chondrogenesis, the PAC1 receptor is expressed in chondroprogenitor and chondroid cells, suggesting that PACAP regulates extravascular chondrogenic and osteogenic differentiation and cartilage development [41, 42, 46]. Furthermore, PACAP has been shown to protect articular cartilage from degeneration and to reduce oxidative stress-induced matrix degradation in cultured chondrocytes [47, 48]. Thus, it seems reasonable to assume that similar mechanisms are involved in atherosclerosis-associated vascular chondrogenesis.

Our findings raise the question of whether the extracellular matrix (ECM), synthesized by chondrocytes [49], is involved in vascular chondrogenesis as reported for non-vascular chondrogenesis. Non-vascular ECM plays critical regulatory roles and orchestrates cell signaling, functions, and morphology of chondrocytes and other cells [50]. Alcian blue identifies ECM proteoglycans and is frequently used to detect chondrogenesis in cartilaginous tissues [51]. Here, we successfully used alcian blue staining to determine and localize vascular chondrogenesis in atherosclerotic lesions in BT of ApoE^−/−^ mice with PAC1 or PACAP deficiency under SC or CED showing a significant reduction of the alcian blue positive areas in atherosclerotic lesions in BT of PAC1^−/−^/ApoE^−/−^ compared to ApoE^−/−^ and PACAP^−/−^/ ApoE^−/−^mice after 20 weeks CED, but not after SC. This observation indicates for the first time that PAC1 deficiency in ApoE^−/−^ mice causes a reduction of ECM proteoglycans under pro-atherogenic conditions. As alcian blue allows the detection of presumptive cartilage formation, the Alcian blue stained area may represent a precursor cartilage-forming core, as described by others [52]. Collagen II expression is a hallmark of chondrocyte differentiation, which is crucial in determining plaque stability [53]. Cartilage ECM is composed primarily of collagen II expression, a hallmark of chondrogenesis, e.g., in the aorta [51, 52].

To further characterize alcian blue-positive areas in lesions, we investigated the presence of collagen II because its content in human arteries relates to atherosclerosis and calcification stage [54]. Our results revealed a significant correlation between proteoglycans, alcian blue positive areas and collagen II IR in atherosclerotic plaques, but not in the tunica media after SC or CED.

At present, it remains unclear (1) why the anti-chondrogenic effect of PAC1 deficiency was only seen under CED and not under SC; (2) why this effect was independent of plasma cholesterol or triglyceride levels in comparison with ApoE^−/−^ and PACAP^−/−^/ApoE^−/−^ mice under CED or SC, respectively. Also, our previously published results revealed that neither PAC1 nor PACAP lacks in ApoE^−/−^ mice modify lipidemia under CED feeding [39, 40]. Although our present manuscript includes results from another group of mice in comparison with previous publications [39, 40], we observed similar lipidemia levels as described earlier. Thus, we speculate that the anti-chondrogenic effect of PAC1 deficiency in ApoE^−/−^ mice is only operational at a distinct elevated cholesterol/triglyceride level.

Therefore, it is reasonable to suggest that exogenous PAC1 receptor antagonists may have anti-atherogenic potential under a high-fat diet in animal models and humans with highly elevated blood lipid levels, like hypercholesterolemia.

In this context, we have previously found that PAC1^−/−^/ApoE^−/−^ animals with reduced lumen stenosis compared to ApoE^−/−^ mice after 20 wks CED, also have a significant reduction of collagen inside atherosclerotic lesions [40], which is also in agreement with the results presented here. Consequently, it can be expected that agonizing the PACAP binding receptors or antagonizing PAC1 could become an appropriate target for atherosclerosis treatment by reducing plaque-associated chondrogenesis and may induce the regression of the lesions. Thus, future experiments are necessary to confirm this by treatment of mice with stable PACAP agonists and selective PAC1, VPAC1, or VPAC2 agonists and antagonists. Moreover, to definitively decipher the receptor-specific mechanisms, it will be necessary to investigate the effects of these agonists/antagonists on atherosclerosis development and progression and plaque associated chondrogenesis in ApoE-/- mice of single or double VPAC1- and/or VPAC2-deficiencies in these animal models. In the present study, we additionally tested the influence of PACAP and PAC1 deficiencies in ApoE^−/−^ mice under CED and SC on the cellular plasticity and molecular signatures associated with chondrogenesis in the atherosclerotic vascular wall. Our data suggest the following: PAC1 deficiency associated with anti-chondrogenic signatures can be due to the PAC1-dependent transformation of smooth muscle cells (SMC) and MΦ into foam cells, as well as to their participation in the calcification process of the arterial wall of atherosclerotic lesions. This can help to unravel PACAP signaling-dependent mechanistic factors of the PAC1 deficiency-driven anti-chondrogenic effect. Such phenomena have been described previously [16, 25, 55]. In this regard, cartilage-like areas within the media, which stain positive for alcian blue and collagen II, show no immunoreactivity for sm-*α-*actin, whereas neighboring regions free from the cartilage-like matrix are sm-*α-*immunoreactive. These observations underline that SMCs lose their classic markers, transdifferentiate into chondrocyte-like cells, and induce matrix mineralization via chondrogenic transcription pathways (reviewed by [16]. However, SMCs and MΦ might also convert into foam cells [56]. SMCs can, for example, increase the RUNX2 mRNA level and, thus, mediate a conversion into osteoblast-like cells [56]. In any case, we propose that PAC1 signaling is involved in these processes.

We investigated the influence of PACAP and PAC1 deficiency on RUNX2 immunoreactivities in the atherosclerotic vessel wall, because chondrogenesis and osteogenesis are known to be positively regulated by the transcription factor RUNX2 and influenced by VIP/ PACAP signaling [30, 42, 57–59]. Paradoxically, we found an increase of RUNX2 expression in atherosclerotic plaques of PAC1^−/−^ApoE^−/−^ mice under CED compared to PACAP^−/−^ApoE^−/−^ and a positive and significant correlation between RUNX2 and SMC after 20 weeks of CED but not with Iba1 MΦ a marker used to detect MΦ in atherosclerotic lesions [60].

We further addressed the question of how chondrogenesis-associated inflammatory signatures in the vascular wall are influenced by PACAP/PAC1 deficiency and cholesterol feeding. In our study, PAC1 deficiency significantly increases the percentage of MΦ area in atherosclerotic plaques compared with PACAP^−/−^ApoE^−/−^ mice only under SC. Our data suggest that PACAP and PAC1 regulate chondrogenesis related to atherogenesis in SMC, but not in MΦ. Interestingly, this effect was observed only under SC; this may indicate that a part of the MΦ in atherosclerotic lesions of PAC1^−/−^ApoE^−/−^ mice did not differentiate into giant foam cells; consequently, the high percentage of Iba1 immunoreactive MΦ observed in plaques of PAC1 deficient mice indicates that the lack of PAC1 did not inhibit their (foam cell) formation. This may be because PAC1 deficiency did not affect the lumen stenosis after SC or 10 weeks CED, as described previously [40]. However, inflammatory processes do not appear to be involved since IL-1β, IL-6, TNF-α and COX-2 immunoreactivities are not affected by the lack of PAC1 in ApoE^−/−^ animals after 20 weeks of CED [40]. In contrast after 30 weeks SC, IL-1β and TNF were reduced compared to ApoE^−/−^ mice [40]. Moreover, our investigations show subpopulations of foam cells that differ in their Iba1-immunoreactivity (~ MΦ) and are, in part, RUNX2-immunoreactive. Studies by others have described that MΦ expresses bone matrix proteins such as osteopontin, osteocalcin, and alkaline phosphatase and are involved in the regulation of tissue mineralization [11, 15]. Our investigations also indicate that MΦ can transdifferentiate into a phenotype involved in the regulation of calcification and chondrogenesis within atherosclerotic lesions. SMCs and MΦ may be involved in atherosclerosis-associated chondrogenesis due to phenotypic cellular changes but with different pathways. However, the mechanistic involvement of PACAP signalling in these processes remains to be clarified further.

## Summary and conclusions

Taken together, our present data demonstrate that inhibition of chondrogenesis by PAC1 deficiency in ApoE^−/−^ mice is an additional atheroprotective effect to the alleviation of the development and progression of atherosclerotic plaques by reduction of the lumen stenosis as described previously [40]. The anti-chondrogenic effect of PAC1 deficiency seems to occur via modification of the extracellular matrix of atherosclerotic lesions and the transformation of SMCs into chondrocytes involved in this process.

Our findings open therapeutic perspectives of highly selective PAC1 antagonists, or PACAP agonists, to limit chondrogenesis and atherogenesis, especially under high-fat diet or in humans with highly elevated blood lipid levels.

### Limitations

Although we could provide compelling evidence that PACAP signalling is involved in atherogenesis associated chondrogenesis especially after high fat diet the precise cellular and molecular mechanisms in the vascular wall remained unclear.

## Data Availability

The datasets that support the findings of this study are available from the corresponding author upon reasonable request.

## Abbreviations

ADCYAP1: adenylate cyclase activating polypeptide 1
ApoE: apolipoprotein E
ApoE−/−: ApoE deficiency
BT: brachiocephalic trunk
CED: cholesterol-enriched diet
DNA: deoxyribonucleic acid
HE: hematoxylin-eosin
Iba1: ionized Calcium-Binding Adapter Molecule 1
IR: immunoreactivity
MΦ: macrophage
oxLDL: oxidized low-density lipoprotein
PAC1: pituitary adenylate cyclase-activating polypeptide type I receptor
PACAP: pituitary adenylate cyclase activating polypeptide 1
PBS: phosphate-buffered saline
PCR: polymerase chain reaction
RUNX2: RUNX Family Transcription Factor 2
SC: standard chow
SEM: standard error of the mean
SMC: smooth muscle cells
VIP: vasoactive intestinal polypeptide
VPAC1: vasoactive intestinal polypeptide receptor 1
VPAC2: vasoactive intestinal polypeptide receptor 2
